# Sex Differences in Heart Failure Epidemiology and Clinical Characteristics in Spain: A Nationwide Population-Based Study

**DOI:** 10.3390/jcm15134879

**Published:** 2026-06-23

**Authors:** Andrea Severo, Diego Alvaredo Rodrigo, Javier González Martín, Sonia Rivas García, Irene Marco, Beatriz Palacios, Victoria González, Margarita Capel, Javier de Juan Bagudá, Fernando Arribas Ynsaurriaga, María Dolores García-Cosío Carmena, Juan Francisco Delgado Jiménez

**Affiliations:** 1 Cardiology, Hospital Universitario 12 de Octubre, Instituto de Investigación Sanitaria Hospital 12 de Octubre (imas12), Centro de Investigación Biomédica en Red de Enfermedades Cardiovasculares (CIBERCV), 28041 Madrid, Spain; javier.gonzalezm@salud.madrid.org (J.G.M.); soniarivas.imas12@h12o.es (S.R.G.); javier.juan@salud.madrid.org (J.d.J.B.); fernando.arribas@salud.madrid.org (F.A.Y.); mariadolores.garcia-cosio@salud.madrid.org (M.D.G.-C.C.); juan.delgado@salud.madrid.org (J.F.D.J.); 2 Faculty of Medicine, Universidad Complutense de Madrid, 28040 Madrid, Spain; 3 Instituto de Investigación Sanitaria Hospital 12 de Octubre (imas12), 28041 Madrid, Spain; diealv.imas12@h12o.es; 4 Cardiology, Hospital Clínico San Carlos, 28040 Madrid, Spain; irene.marco@salud.madrid.org; 5 BioPharmaceuticals Medical, AstraZeneca, 28050 Madrid, Spain; beatriz.palacios@astrazeneca.com (B.P.); victoriaisabel.gonzalez@astrazeneca.com (V.G.); 6 BioPharmaceuticals Corporate Affairs & Market Access, AstraZeneca, 28050 Madrid, Spain; margarita.capel@astrazeneca.com; 7 Faculty of Medicine, Health and Sports, Department of Medicine, Universidad Europea, 28670 Madrid, Spain

**Keywords:** heart failure, epidemiology, sex factors, ventricular function, left

## Abstract

**Background:** Heart failure (HF) is a major public health problem and a paradigmatic condition for sex differences in cardiovascular disease. However, national population-based evidence describing these differences remains limited. We aimed to provide the first nationwide sex-stratified epidemiologic characterization of HF in Spain, quantifying incidence, prevalence, and clinical characteristics across age groups and left ventricular ejection fraction (LVEF) categories. **Methods:** We conducted a retrospective population-based study using the BIG-PAC database, integrating electronic health records from primary and hospital care covering approximately 1.8 million individuals across seven Spanish autonomous communities. Adult patients with incident HF between 2013 and 2019 were identified. HF phenotypes were classified according to LVEF as reduced (HFrEF ≤40%), mildly reduced (HFmrEF 41–49%), preserved (HFpEF ≥50%), or unknown (HFuEF). Incidence rates per 1000 person-years and prevalence were estimated and stratified by sex and LVEF phenotype. **Results:** In total, 19,961 incident HF cases were identified. Overall HF incidence was 3.23 per 1000 person-years and was similar in women and men (*p* = 0.697). HF prevalence was 2.34% and higher in men than in women (2.67% vs. 2.06%; *p* < 0.001). Women were older and more frequently presented with HFpEF (38%), whereas HFrEF predominated in men (53%); notably, HFrEF still accounted for approximately one third of HF cases among women. Once stratified by LVEF phenotype, clinical characteristics were broadly similar between sexes. **Conclusions:** While HF incidence was similar in women and men, substantial sex differences in prevalence, age, and phenotype distribution were identified, establishing the first nationwide epidemiological framework to inform sex-aware HF prevention and healthcare planning in Spain.

## 1. Introduction

Heart failure (HF) remains one of the leading causes of morbidity, mortality, and healthcare expenditure worldwide. Recent European analyses reveal substantial geographic variability in HF epidemiology and outcomes, underscoring the need for high-quality, country-specific data to inform prevention strategies and health system planning [1].

HF exemplifies a medical condition with pronounced sex differences across the entire disease continuum—from epidemiology and risk factors to clinical expression and prognosis [2]. Despite increasing scientific attention to sex- and gender-related differences, the available evidence remains heterogeneous, frequently derived from selected populations, and often insufficiently robust [2,3,4]. In Spain, several studies have delineated the population burden and healthcare implications of HF within the country’s universal health system [5,6,7], yet none have specifically evaluated HF epidemiology through a sex-stratified lens. Consequently, the prevalence, incidence, and clinical profile of HF in women and men at the national level remain poorly defined.

BIG-PAC is a longitudinal, population-based database including approximately 1.8 million individuals from seven Spanish autonomous communities since 2012 and has been extensively used in large-scale epidemiological studies of HF in Spain [5,6,7]. Previous demographic validation analyses comparing BIG-PAC with Spanish national census data demonstrated close agreement in age and sex distributions between the BIG-PAC population and the overall Spanish population, supporting its use as a representative source for nationwide epidemiological research [5]. The database integrates electronic health records from primary care centers and referral hospitals within the Spanish National Health System. Using this platform, the present study provides the first nationwide, sex-specific epidemiologic characterization of HF in Spain, quantifying incidence, prevalence, and baseline clinical features across age groups and left ventricular ejection fraction (LVEF) categories. This evidence is essential to support sex-informed prevention strategies, refine risk stratification, and guide health system planning within a universal public healthcare framework.

**Figure 1:**
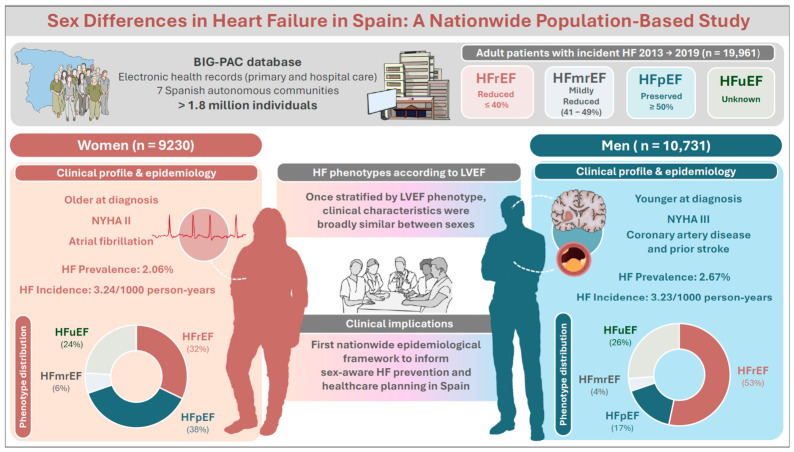
Central illustration. Graphical abstract summarizing the design and main findings of this nationwide population-based study, including sex-specific differences in heart failure incidence, prevalence, clinical characteristics, and distribution of LVEF phenotypes. AF = Atrial fibrillation, HF = Heart failure, HFmrEF = Heart failure with mildly reduced ejection fraction, HFpEF = Heart failure with preserved ejection fraction, HFrEF = Heart failure with reduced ejection fraction, HFuEF = Heart Failure with unknown ejection fraction, LVEF = Left ventricular ejection fraction, NYHA = New York Heart Association.

A graphical summary of the study design and main findings is provided in the central illustration (Figure 1).

## 2. Methods

### 2.1. Study Design and Data Source

This is a retrospective, longitudinal, population-based study of sex-stratified epidemiological estimates and baseline clinical characteristics derived from the BIG-PAC database (Atrys Health, Madrid, Spain), following the methodological framework of previously published nationwide HF studies in Spain [5,6,7].

BIG-PAC operates under a dissociated data model and is registered with the European Medicines Agency. Data are available from 2012 onwards and are updated on a monthly basis, ensuring continuous capture of clinical information from primary and hospital care. All data are irreversibly anonymized prior to access, ensuring compliance with Spanish and European data protection regulations; neither the study sponsor nor the investigators had access to identifiable patient-level data. The study protocol was approved by the Clinical Research Ethics Committee of Hospital Universitario 12 de Octubre (Madrid, Spain; CEIm no. 25/616).

### 2.2. Study Population and Variables

Adult patients aged ≥18 years with at least 1 year of continuous registration in the database prior to the index date were eligible for inclusion. HF was identified by at least one new diagnosis coded according to the International Classification of Diseases, Ninth or Tenth Revision (ICD-9-CM/ICD-10-CM), recorded in inpatient or outpatient care between 1 January 2013 and 30 September 2019, consistent with previous nationwide studies using the BIG-PAC database [5,6,7]. The index date was defined as the date of the first recorded HF diagnosis. Patients with chronic stage V kidney disease requiring dialysis at any time before the index date were excluded.

HF phenotypes were classified according to LVEF, when available: HF with preserved LVEF (HFpEF) was defined as LVEF ≥ 50%, HF with reduced LVEF (HFrEF) as LVEF ≤ 40%, HF with mildly reduced LVEF (HFmrEF) as LVEF > 40% and <50%, and HF with unknown LVEF (HFuEF) as the absence of echocardiographic data. Baseline demographic characteristics, cardiovascular risk factors, and other clinically relevant comorbidities routinely captured in electronic health records were recorded and described for the overall HF population and stratified by sex, age group, and LVEF phenotype.

### 2.3. Statistical Analysis

Baseline clinical characteristics were summarized using descriptive statistics. Continuous variables were presented as mean and standard deviation, and categorical variables as absolute numbers and percentages, overall and by sex. Analyses were performed on aggregated data, stratified by sex, calendar year or LVEF phenotype. For the comparisons of continuous variables by sex, a bilateral Welch *t*-test was used. For the comparison of dichotomous variables by sex, proportion differences were tested, and for polytomous variables a single global *p*-value from global *Χ*^2^ contrast was reported.

HF incidence rates were calculated as the number of newly diagnosed HF cases during the study period divided by the total person-time at risk contributed by adult individuals without prevalent HF and are expressed per 1000 person-years. HF prevalence was estimated as the proportion of individuals living with HF in each calendar year relative to the adult population alive and enrolled at the beginning of the calendar year, with continuous enrollment during the entire prior year in the database. Incidence and prevalence estimates were computed overall and stratified by sex, age group, and LVEF phenotype and are reported with corresponding 95% confidence intervals (CI), following the methodological approach used in prior nationwide BIG-PAC studies. Incidence and prevalence by year were compared between men and women: for incidence, observed counts over exposure times were compared bilaterally as Poisson rates; for prevalence, proportions of cases over the population at risk were compared. Trends over time were analyzed with log-linear models that estimated the annual percent change and 95% CI, overall and by sex. The *p*-value of an interaction term fitted into the models provided the evidence for differences in time trends by sex. A two-sided *p*-value <0.05 was considered statistically significant. The reported *p*-values should be interpreted with caution, as they have not been adjusted for potential confounding factors or for multiplicity (multiple comparisons). All analyses were performed using R Statistical Software (version 4.5.1).

## 3. Results

### 3.1. Heart Failure Incidence

**Figure 2:**
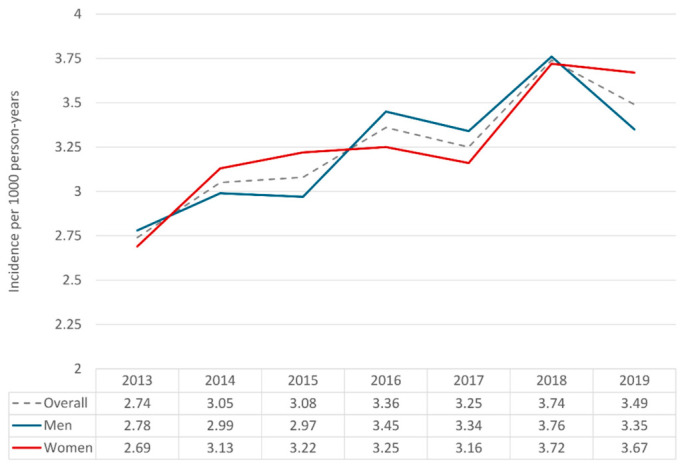
Heart failure incidence by sex in Spain, 2013–2019. Annual incidence rates per 1000 person-years for the overall population, men, and women. Data for 2019 include January to September.

The overall HF incidence was 3.23 per 1000 person-years and increased over time, rising from 2.74 in 2013 to 3.74 in 2018 (Figure 2). Estimates for 2019 reflect partial-year data.

Overall HF incidence was virtually identical in women and men (*p* = 0.697), with annual rates following parallel temporal trends throughout the study period (Figure 2). When stratified by LVEF, marked sex-specific patterns emerged. HFrEF incidence was higher in men, whereas HFpEF and HFmrEF incidence rates were higher in women (*p* < 0.001). Complete annual estimates by sex, including 95% CI and *p* values for sex comparisons across LVEF categories, are provided in Supplementary Table S1.

### 3.2. Heart Failure Prevalence

**Figure 3:**
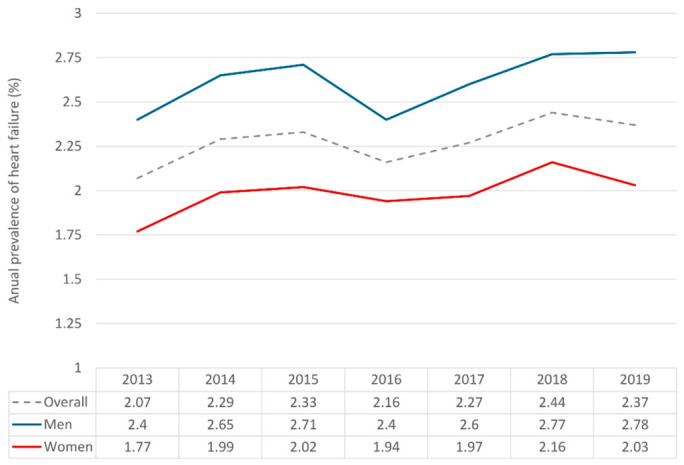
Heart failure prevalence by sex in Spain, 2013–2019. Annual prevalence (%) in the overall population, men, and women. Data for 2019 include January to September.

The overall prevalence of HF in the adult BIG-PAC population was 2.34% (43,578 of 1,865,061 individuals) and showed a gradual increase over the study period. HF prevalence was consistently higher in men than in women. Overall, 2.67% of men and 2.06% of women were living with HF (*p* < 0.001). This sex difference was observed across all calendar years, with parallel temporal patterns in both sexes (Figure 3).

**Figure 4:**
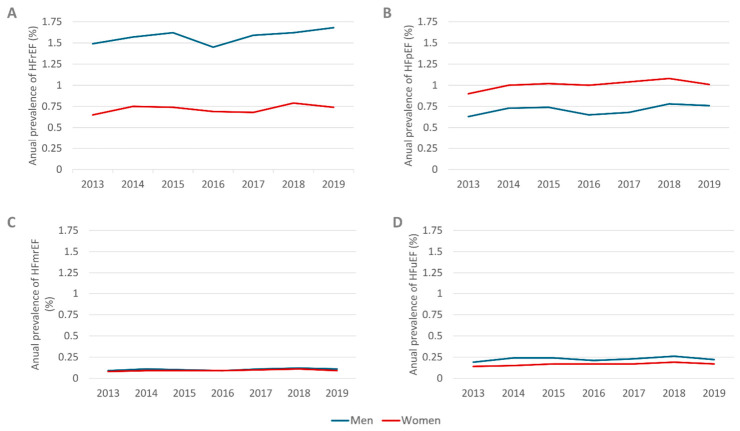
Sex-specific trends in heart failure prevalence by left ventricular ejection fraction category in Spain (2013–2019). Temporal trends in heart failure prevalence stratified by sex across left ventricular ejection fraction categories: (**A**) HFrEF, (**B**) HFpEF, (**C**) HFmrEF, and (**D**) HFuEF. All panels share the same Y-axis scale to facilitate comparison across phenotypes. Data for 2019 include January to September. HF = Heart failure, HFmrEF = Heart failure with mildly reduced ejection fraction, HFpEF = Heart failure with preserved ejection fraction, HFrEF = Heart failure with reduced ejection fraction, HFuEF = Heart Failure with unknown ejection fraction.

When stratified by LVEF, marked sex-related differences in HF prevalence were observed. HFrEF predominated in men, whereas HFpEF was more frequent in women (*p* < 0.001). HFmrEF accounted for a small proportion of cases in both sexes, with only minimal differences between women and men over time. HFuEF represented approximately one quarter of HF cases and remained relatively stable over time in both sexes, with a slightly higher prevalence in men than in women throughout the study period (*p* < 0.001). These sex-specific prevalence patterns across LVEF categories and over time are summarized in Figure 4 and Supplementary Table S2.

### 3.3. Demographic and Clinical Characteristics

Baseline demographic and clinical characteristics at the time of HF diagnosis were analyzed in the incident HF cohort, comprising 19,961 patients. Women were slightly older than men at the time of HF diagnosis (70.6 ± 18.9 vs. 68.9 ± 19.1 years), with a higher proportion of patients aged ≥85 years, whereas younger-onset HF (<45 years) was more frequent among men. Functional status at diagnosis also differed by sex, with women more frequently classified as New York Heart Association (NYHA) class II and men more frequently classified as NYHA class III (Table 1).

HF phenotype distribution differed markedly by sex. Among men, HFrEF was the predominant phenotype (53.3%), whereas among women HFpEF was most frequent (37.6%). Importantly, HFrEF accounted for 32.1% of cases among women with HF, while HFpEF represented 16.5% of cases among men with HF. HFmrEF was uncommon in both sexes, and LVEF was not classified at diagnosis in approximately one quarter of patients (26.2% in men and 23.9% in women) (Table 1).

Most cardiovascular and non-cardiovascular comorbidities showed similar prevalence in women and men; however, coronary artery disease and prior stroke were more frequent in men, whereas atrial fibrillation was more prevalent in women. Although overall comorbidity burden, assessed by the Charlson Comorbidity Index, was statistically higher in men, the absolute difference was small and unlikely to be clinically meaningful (Table 1).

### 3.4. Heart Failure with Reduced Ejection Fraction

Among patients with HFrEF, age at diagnosis and age distribution were similar in women and men. NYHA class III was the most frequent functional class in both sexes. Notably, neither comorbidity patterns nor overall comorbidity burden differed meaningfully by sex, even for coronary artery disease, which showed a comparable prevalence in women and men (Table 2).

### 3.5. Heart Failure with Preserved Ejection Fraction

Patients with HFpEF were older at diagnosis than those with HFrEF, irrespective of sex, and age distributions within HFpEF were highly similar between women and men, with a high proportion of very elderly patients. NYHA class II predominated at diagnosis in both sexes. Most cardiovascular and non-cardiovascular comorbidities showed a broadly similar distribution in women and men (Table 3).

### 3.6. Heart Failure with Mildly Reduced and Unknown Ejection Fraction

In patients with HFmrEF, demographic and clinical characteristics were broadly similar between women and men and were more closely aligned with those observed in HFpEF; detailed results are provided in the Supplementary Table S3. Similarly, in patients with HFuEF, these characteristics did not differ meaningfully by sex and were predominantly observed in older age groups; detailed results are presented in Supplementary Table S4.

## 4. Discussion

HF is a major global public health problem and represents a paradigmatic condition for sex differences in cardiovascular disease. However, despite growing awareness of sex- and gender-related disparities, available evidence remains heterogeneous and is largely derived from selected cohorts or clinical trials with limited external validity and persistent underrepresentation of women [1,2,3,4].

In this context, our nationwide population-based study provides the first comprehensive sex-specific characterization of HF epidemiology in Spain, integrating incidence, prevalence, and baseline clinical characteristics across LVEF categories using a large, representative real-world database. Although previous Spanish studies have described HF burden and healthcare utilization, sex has not been examined as the primary analytical axis at the national level, leaving key epidemiological questions unanswered [5,6,7].

In our cohort, overall HF incidence was virtually identical in women and men, and both the overall rates and the LVEF-stratified sex-specific patterns were consistent with contemporary population-based data from Europe and North America [1,8,9]. In the recent European HF Survey, the median incidence of HF across participating countries was approximately 3.9 cases per 1000 person-years, while population-based studies from North America have reported incidence estimates generally ranging between about 2 and 5 cases per 1000 person-years, closely overlapping with the incidence observed in our cohort [1,8,9].

HF prevalence was higher in men than in women, closely aligning with contemporary European and United States population-based estimates [1,8,9]. Across Western populations, overall HF prevalence is typically reported between approximately 1% and 3% of the adult population and is generally slightly higher in men than in women, with sex differences largely reflecting the higher burden of HFrEF among men and the greater representation of HFpEF among women [1,8,9,10]. As previously reported, this male predominance coexists with a higher proportion of HFpEF among women, with sex differences in prevalence tending to attenuate at older ages [1,8,9,10].

However, direct comparisons between studies should be interpreted cautiously, as epidemiological estimates are strongly influenced by data sources and case ascertainment strategies. Several national analyses rely primarily on hospital discharge databases or administrative claims, which may preferentially capture more severe presentations of HF. In contrast, the present study is based on nationwide electronic health records capturing both ambulatory and hospitalized care, allowing identification of HF across the full spectrum of disease severity in routine clinical practice.

A key contribution of our cohort is the nationwide characterization of women with HFrEF—the main substrate for advanced HF—in terms of prevalence, incidence, and age at diagnosis. Notably, this population accounts for approximately one third of all women with HF, providing essential context to interpret sex differences observed in advanced HF therapies and to determine, in dedicated studies, whether these differences primarily reflect phenotype distribution at disease onset or are influenced by additional factors such as delays or barriers in referral to specialized HF centers [11].

An important and clinically relevant finding was the high proportion of patients without documented LVEF at the time of HF diagnosis. LVEF was unavailable in approximately one quarter of patients, was slightly more frequent in men, and clustered in older age groups; importantly, this proportion did not decline over time. Similar challenges with incomplete LVEF characterization have been recognized in population-based cohorts [1,8,9], with more than 60% of patients lacking documented LVEF in some nationwide studies [8], underscoring persistent limitations in real-world phenotyping rather than a sex-specific access signal and highlighting a clear area for improvement in routine HF assessment.

Rather than excluding these patients, we analyzed HFuEF as a separate category to avoid potential misclassification into established HF phenotypes. Although incomplete LVEF characterization may have influenced the precise distribution of phenotype-specific estimates, the similar prevalence of HFuEF in women and men and the absence of meaningful sex-related differences within this subgroup suggest that this limitation is unlikely to have materially affected the principal sex-specific findings of the present study.

Overall, most observed sex differences in HF presentation appear to reflect differences in age at diagnosis and LVEF phenotype distribution rather than intrinsically distinct clinical profiles. Women were older at diagnosis and more frequently presented with HFpEF, whereas men more commonly exhibited HFrEF. Beyond these differences, once stratified by LVEF, clinical characteristics—including comorbidity burden, comorbidity patterns, and functional status—were broadly overlapping between women and men.

This pattern is consistent with prior mechanistic and epidemiological evidence suggesting that sex-related differences in HF arise from long-term trajectories of cardiac remodeling and vascular aging, shaped by cumulative lifetime exposure to general and sex-specific cardiovascular risk factors, with contributions from inflammatory pathways and modulation by sex hormones. Women more frequently develop HF in association with hypertension, obesity, atrial fibrillation, and diastolic dysfunction, whereas men more commonly present with ischemic heart disease and adverse ventricular remodeling, factors that may contribute to the predominance of HFpEF and HFrEF, respectively [2,3,4,10]. However, the present study was not designed to investigate underlying biological mechanisms; therefore, these pathways should be interpreted as conceptual frameworks rather than causal explanations of the clinical patterns observed at diagnosis.

At the population level, these differences in HF phenotype distribution have direct implications for prevention strategies, diagnostic pathways, healthcare organization, and resource allocation within a universal public health system, supporting a shift toward phenotype-, age-, and sex-oriented care models rather than a uniform approach to HF management. In particular, the higher proportion of HFpEF among women highlights the need to strengthen diagnostic strategies for this phenotype in routine clinical practice. HFpEF in women may present with less specific symptoms and frequently overlaps with common comorbidities such as obesity, hypertension, and atrial fibrillation, which may delay recognition. Accordingly, improved diagnostic pathways—including systematic biomarker-based assessment together with structured echocardiographic evaluation—may facilitate earlier identification and more accurate phenotyping, particularly in women. Importantly, as disease-modifying therapies for HFpEF are now available, improving diagnostic accuracy has direct implications for timely initiation of effective treatment.

Several limitations should be acknowledged. The retrospective observational design based on routinely collected healthcare data is inherently subject to misclassification and under-recording. LVEF was not available for all patients at the time of HF diagnosis, which may have introduced information bias in phenotype classification. Furthermore, the database includes individuals who interact with the healthcare system and may not fully capture undiagnosed cases or those with limited access to medical care; therefore, differences in healthcare-seeking behavior or diagnostic assessment between sexes cannot be excluded. Residual confounding by unmeasured variables, including socioeconomic factors, is also possible. In addition, the analyses were descriptive and no multivariable adjustment was performed; therefore, the observed sex differences should not be interpreted as independent associations attributable to sex itself. Given the large sample size of this population-based study, some variables reached statistical significance despite small absolute differences that are unlikely to be clinically meaningful. Accordingly, findings should be interpreted in terms of their magnitude and clinical relevance. While full results are presented in the tables and Supplementary Material, the main text focuses on those differences with greater magnitude or clinical relevance.

Despite these limitations, the nationwide scope, large sample size, and consistency of our findings with international population-based series support the robustness and external validity of the results.

## 5. Conclusions

This nationwide population-based study provides the first comprehensive sex-specific characterization of HF epidemiology in Spain. While overall HF incidence was similar in women and men, clear sex differences were observed in HF prevalence, age at diagnosis, and phenotype distribution. These findings provide a robust epidemiological framework to inform prevention strategies, guide healthcare planning within a universal public health system, and support future analyses of sex- and phenotype-specific patterns of clinical management and outcomes.

## Figures and Tables

**Table 1:** Baseline characteristics of the incident heart failure cohort, overall and stratified by sex (2013–2019).

	HF Incident Cohort (N = 19,961)	Men (N = 10,731)	Women (N = 9230)	*p* Value
**Age at index date (years)**	69.7 ± 19.0	68.9 ± 19.1	70.6 ± 18.9	<0.001
**Age groups**				<0.001
<45	13.3%	14.3%	12.2%	
45–64	28.6%	29.2%	27.9%	
65–74	14.2%	14.2%	14.2%	
75–84	14.8%	15.2%	14.3%	
≥85	29.1%	27.2%	31.4%	
**NYHA class at index date**				<0.001
I	13.4%	13.5%	13.3%	
II	41.0%	38.8%	43.6%	
III	41.5%	43.3%	39.3%	
IV	2.8%	3.0%	2.5%	
Unknown	1.4%	1.5%	1.3%	
**LVEF category**				<0.001
HFrEF	43.5%	53.3%	32.1%	
HFpEF	26.3%	16.5%	37.6%	
HFmrEF	5.1%	4.0%	6.4%	
HFuEF	25.1%	26.2%	23.9%	
**Charlson Comorbidity Index**	2.7 ± 1.6	2.9 ± 1.9	2.7 ± 1.6	<0.001
**Cardiovascular comorbidities**				
**Hypertension**	59.1%	59.2%	59.0%	0.749
**Dyslipidaemia**	44.9%	44.7%	45.1%	0.581
**Diabetes type 1**	3.7%	3.8%	3.6%	0.618
**Diabetes type 2**	27.6%	27.8%	27.4%	0.561
**Atrial fibrillation**	28.2%	27.2%	29.5%	<0.001
**Coronary artery disease**	33.1%	34.6%	31.3%	<0.001
**Peripheral arterial disease**	4.7%	4.9%	4.6%	0.314
**Other comorbidities**				
**Stroke**	10.1%	10.9%	9.2%	<0.001
**Chronic kidney disease**	26.7%	27.8%	25.5%	0.745
**Stage unknown**	11.1%	11.6%	10.6%	
**Stage I**	0.6%	0.6%	0.7%	
**Stage II**	2.8%	2.9%	2.7%	
**Stage III**	9.2%	9.6%	8.6%	
**Stage IV**	2.2%	2.3%	2.1%	
**End stage**	1.0%	0.9%	0.8%	
**COPD**	13.2%	13.6%	12.8%	0.086
**Asthma**	8.9%	9.1%	8.7%	0.333
**Anemia**	26.0%	25.6%	26.5%	0.115
**Hepatic disease**	4.8%	5.0%	4.6%	0.146
**Malignant neoplasm**	11.9%	11.9%	11.9%	0.961

Abbreviations: COPD = Chronic obstructive pulmonary disease, NYHA = New York Heart Association, LVEF = Left ventricular ejection fraction, HF = Heart failure, HFmrEF = Heart failure with mildly reduced ejection fraction, HFpEF = Heart failure with preserved ejection fraction, HFrEF = Heart failure with reduced ejection fraction, HFuEF = Heart Failure with unknown ejection fraction.

**Table 2:** Baseline characteristics of patients with incident heart failure and reduced left ventricular ejection fraction, stratified by sex (2013–2019).

	HFrEF Cohort (N = 8678)	HFrEF Men (N = 5719)	HFrEF Women (N = 2959)	*p* Value
**Age at index date (years)**	65.6 ± 18.6	65.4 ± 18.6	66.0 ± 18.7	0.156
**Age groups**				0.321
<45	17.9%	18.3%	17.1%	
45–64	31.3%	31.4%	31.1%	
65–74	15.1%	15.2%	15.1%	
75–84	16.0%	16.0%	15.9%	
≥85	19.7%	19.1%	20.9%	
**NYHA class at index date**				0.306
I	13.3%	13.2%	13.7%	
II	35.0%	34.4%	36.3%	
III	46.5%	47.3%	44.9%	
IV	3.8%	3.8%	3.7%	
Unknown	1.4%	1.4%	1.5%	
**Charlson Comorbidity Index**	2.8 ± 1.6	3.0 ± 2.0	2.6 ± 1.5	<0.001
**Cardiovascular comorbidities**				
**Hypertension**	61.0%	60.7%	61.6%	0.424
**Dyslipidaemia**	43.9%	43.1%	45.5%	0.036
**Diabetes type 1**	4.1%	4.1%	4.0%	0.785
**Diabetes type 2**	28.3%	28.2%	28.5%	0.781
**Atrial fibrillation**	23.5%	24.0%	22.6%	0.155
**Coronary artery disease**	38.7%	38.7%	38.9%	0.853
**Peripheral arterial disease**	5.1%	5.1%	5.0%	0.888
**Other comorbidities**				
**Stroke**	12.3%	12.1%	12.6%	0.513
**Chronic kidney disease**	30.8%	30.9%	30.6%	0.469
**Stage unknown**	13.0%	12.7%	13.5%	
**Stage I**	0.7%	0.7%	0.8%	
**Stage II**	3.1%	3.2%	3.0%	
**Stage III**	10.5%	10.8%	9.9%	
**Stage IV**	2.5%	2.5%	2.4%	
**End stage**	1.1%	1.2%	0.9%	
**COPD**	14.7%	14.6%	14.9%	0.690
**Asthma**	9.5%	9.7%	9.2%	0.472
**Anemia**	25.9%	25.3%	26.9%	0.103
**Hepatic disease**	5.4%	5.4%	5.4%	0.952
**Malignant neoplasm**	12.4%	12.7%	11.8%	0.237

Abbreviations: COPD = Chronic obstructive pulmonary disease, NYHA = New York Heart Association, HF = Heart failure, HFpEF = Heart failure with preserved ejection fraction, HFrEF = Heart failure with reduced ejection fraction.

**Table 3:** Baseline characteristics of patients with incident heart failure and preserved left ventricular ejection fraction, stratified by sex (2013–2019).

	HFpEF Cohort (N = 5244)	HFpEF Men (N = 1772)	HFpEF Women (N = 3472)	*p* Value
**Age at index date (years)**	73.4 ± 18.6	73.5 ± 18.8	73.3 ± 18.5	0.714
**Age groups**				0.481
<45	9.3%	9.4%	9.2%	
45–64	25.7%	26.2%	25.5%	
65–74	13.2%	12.2%	13.7%	
75–84	13.7%	13.1%	13.9%	
≥85	38.2%	39.1%	37.7%	
**NYHA class at index date**				0.830
I	12.8%	12.9%	12.7%	
II	51.6%	51.4%	51.6%	
III	33.4%	33.4%	33.4%	
IV	1.4%	1.2%	1.4%	
Unknown	1.0%	1.1%	0.9%	
**Charlson Comorbidity Index**	2.5 ± 1.5	2.6 ± 1.5	2.5 ± 1.5	0.022
**Cardiovascular comorbidities**				
**Hypertension**	56.2%	55.2%	56.7%	0.294
**Dyslipidaemia**	44.0%	45.1%	43.5%	0.261
**Diabetes type 1**	3.2%	3.2%	3.3%	0.855
**Diabetes type 2**	25.8%	25.1%	26.2%	0.367
**Atrial fibrillation**	35.5%	35.4%	35.5%	0.959
**Coronary artery disease**	26.2%	27.4%	25.6%	0.163
**Peripheral arterial disease**	3.2%	2.7%	3.5%	0.146
**Other comorbidities**				
**Stroke**	6.0%	5.8%	6.1%	0.672
**Chronic kidney disease**	22.5%	23.0%	22.3%	0.161
**Stage unknown**	9.3%	9.8%	9.0%	
**Stage I**	0.6%	0.4%	0.7%	
**Stage II**	2.8%	2.4%	2.9%	
**Stage III**	7.7%	7.9%	7.6%	
**Stage IV**	1.7%	2.1%	1.5%	
**End stage**	0.9%	0.3%	0.6%	
**COPD**	11.9%	11.8%	12.0%	0.820
**Asthma**	7.9%	8.6%	7.6%	0.203
**Anemia**	27.7%	27.4%	27.9%	0.713
**Hepatic disease**	5.0%	5.1%	4.9%	0.808
**Malignant neoplasm**	10.8%	9.6%	11.4%	0.042

Abbreviations: COPD = Chronic obstructive pulmonary disease, NYHA = New York Heart Association, HF = Heart failure, HFpEF = Heart failure with preserved ejection fraction.

## Data Availability

The data that support the findings of this study are available from the corresponding author upon reasonable request.

## Supplementary Materials

The following supporting information can be downloaded at: https://www.mdpi.com/article/10.3390/jcm15134879/s1, Table S1. Annual heart failure incidence rates by sex and left ventricular ejection fraction category in Spain (2013–2019); Table S2. Annual heart failure prevalence by sex and left ventricular ejection fraction category in Spain (2013–2019); Table S3. Baseline characteristics of patients with incident heart failure and mildly reduced left ventricular ejection fraction, stratified by sex (2013–2019); Table S4. Baseline characteristics of patients with incident heart failure and unknown left ventricular ejection fraction, stratified by sex (2013–2019).

## Abbreviations

HF	Heart failure
HFrEF	Heart failure with reduced ejection fraction
HFmrEF	Heart failure with mildly reduced ejection fraction
HFpEF	Heart failure with preserved ejection fraction
HFuEF	Heart failure with unknown ejection fraction
LVEF	Left ventricular ejection fraction
