# Supplementary material for: Sex Differences in Heart Failure Epidemiology and Clinical Characteristics in Spain: A Nationwide Population-Based Study
Source: J Clin Med. 2026 Jun 23;15(13):4879. doi: 10.3390/jcm15134879 (PMC13360783; doi:10.3390/jcm15134879)
Supplement: Supplementary file 1 [file jcm-15-04879-s001.zip › jcm-4365391-supplementary.pdf]

Supplementary Table S1. Annual heart failure incidence rates by sex and left ventricular ejection fraction category in Spain (2013–2019).

	Men		Women		P value
	Person years*	Incidence per 1,000 person-years (95% CI)	Person years*	Incidence per 1,000 person-years (95% CI)	
**All HF patients**					
**Overall**	3,346,157	3.23 (3.22 – 3.23)	2,858,128	3.24 (3.24 – 3.25)	0.697
**2013**	524,324	2.78 (2.77 – 2.80)	454,131	2.69 (2.68 – 2.71)	0.405
**2014**	484,394	2.99 (2.97 – 3.01)	390,092	3.13 (3.11 – 3.15)	0.243
**2015**	500,527	2.97 (2.96 – 2.99)	378,135	3.22 (3.21 – 3.24)	0.038
**2016**	492,157	3.45 (3.43 – 3.46)	448,234	3.25 (3.24 – 3.27)	0.105
**2017**	467,663	3.34 (3.32 – 3.36)	446,539	3.16 (3.14 – 3.17)	0.123
**2018**	493,998	3.76 (3.74 - 3.77)	430,623	3.72 (3.71 – 3.74)	0.811
**2019****	383,095	3.35 (3.33 – 3.37)	310,375	3.66 (3.64 – 3.69)	0.029
**HFrEF**					
**Overall**	3,346,157	1.72 (1.72 – 1.73)	2,858,128	1.04 (1.04 – 1.05)	<0.001
**2013**	524,324	1.53 (1.52 – 1.54)	454,131	0.94 (0.93 - 0.95)	<0.001
**2014**	484,394	1.57 (1.56 – 1.58)	390,092	0.90 (0.89 - 0.90)	<0.001
**2015**	500,527	1.58 (1.57 – 1.59)	378,135	1.08 (1.07 – 1.09)	<0.001
**2016**	492,157	1.92 (1.91 – 1.93)	448,234	1.08 (1.08 – 1.09)	<0.001
**2017**	467,663	1.83 (1.81 – 1.84)	446,539	1.09 (1.08 – 1.10)	<0.001
**2018**	493,998	1.84 (1.82 – 1.85)	430,623	1.08 (1.07 – 1.09)	<0.001
**2019****	383,095	1.86 (1.84 – 1.87)	310,375	1.14 (1.13 – 1.15)	<0.001
**HFpEF**					
**Overall**	3,346,157	0.53 (0.53 - 0.53)	2,858,128	1.22 (1.22 – 1.22)	<0.001
**2013**	524,324	0.38 (0.37 - 0.39)	454,131	1.04 (1.03 – 1.05)	<0.001
**2014**	484,394	0.49 (0.49 - 0.50)	390,092	1.29 (1.28 – 1.30)	<0.001
**2015**	500,527	0.51 (0.50 - 0.51)	378,135	1.23 (1.22 – 1.24)	<0.001
**2016**	492,157	0.55 (0.55 - 0.56)	448,234	1.14 (1.14 – 1.15)	<0.001
**2017**	467,663	0.58 (0.57 - 0.58)	446,539	1.10 (1.09 – 1.11)	<0.001
**2018**	493,998	0.65 (0.64 - 0.66)	430,623	1.40 (1.39 – 1.41)	<0.001
**2019****	383,095	0.59 (0.58 - 0.60)	310,375	1.41 (1.40 – 1.42)	<0.001
**HFmrEF**					
**Overall**	3,346,157	0.13 (0.13 - 0.13)	2,858,128	0.21 (0.21 - 0.21)	<0.001
**2013**	524,324	0.09 (0.09 - 0.09)	454,131	0.08 (0.08 - 0.08)	0.664
**2014**	484,394	0.14 (0.13 - 0.14)	390,092	0.22 (0.21 - 0.22)	0.005
**2015**	500,527	0.18 (0.18 - 0.19)	378,135	0.27 (0.26 - 0.27)	0.011
**2016**	492,157	0.08 (0.08 - 0.08)	448,234	0.18 (0.18 - 0.18)	<0.001
**2017**	467,663	0.15 (0.15 - 0.16)	446,539	0.20 (0.20 - 0.21)	0.083
**2018**	493,998	0.16 (0.16 - 0.16)	430,623	0.30 (0.30 - 0.31)	<0.001
**2019****	383,095	0.10 (0.10 - 0.10)	310,375	0.22 (0.22 - 0.23)	<0.001
**HFuEF**					
**Overall**	3,346,157	0.84 (0.84 - 0.84)	2,858,128	0.78 (0.77 - 0.78)	0.005
**2013**	524,324	0.78 (0.77 - 0.79)	454,131	0.63 (0.62 - 0.63)	0.004
**2014**	484,394	0.79 (0.78 - 0.80)	390,092	0.73 (0.72 - 0.74)	0.293
**2015**	500,527	0.71 (0.70 - 0.71)	378,135	0.65 (0.64 - 0.66)	0.343
**2016**	492,157	0.90 (0.89 - 0.90)	448,234	0.85 (0.84 - 0.85)	0.421
**2017**	467,663	0.79 (0.78 - 0.80)	446,539	0.76 (0.75 - 0.77)	0.679
**2018**	493,998	1.11 (1.10 – 1.12)	430,623	0.94 (0.93 - 0.95)	0.012
**2019****	383,095	0.80 (0.80 - 0.81)	310,375	0.89 (0.88 - 0.90)	0.228

CI = Confidence interval, HF = Heart failure, HFmrEF = Heart failure with mildly reduced ejection fraction, HFpEF = Heart Failure with preserved ejection fraction, HFrEF = Heart failure with reduced ejection fraction, HFuEF = Heart Failure with unknown ejection fraction. * Total person time contributed by all adults from the start of the year until the earliest of a qualifying HF diagnosis, death, the study end date, and loss to follow-up. ** Year 2019 includes until September.

Supplementary Table S2. Annual heart failure prevalence by sex and left ventricular ejection fraction category in Spain (2013–2019).

	Men		Women		P value
	Total number of patients*	Prevalence (95% CI) (%)	Total number of patients*	Prevalence (95% CI) (%)	
**All HF patients**					
**Overall**	851,481	2.67 (2.63 - 2.7)%	1,013,580	2.06 (2.03 - 2.09)%	<0.001
**2013**	473,540	2.40 (2.36 - 2.45)%	521,841	1.77 (1.74 - 1.81)%	<0.001
**2014**	401,631	2.65 (2.61 - 2.71)%	500,672	1.99 (1.95 - 2.03)%	<0.001
**2015**	402,861	2.71 (2.66 - 2.76)%	508,757	2.02 (1.98 - 2.06)%	<0.001
**2016**	471,965	2.40 (2.36 - 2.45)%	517,255	1.94 (1.91 - 1.98)%	<0.001
**2017**	456,937	2.60 (2.56 - 2.65)%	514,746	1.97 (1.94 - 2.01)%	<0.001
**2018**	435,791	2.77 (2.73 - 2.82)%	520,670	2.16 (2.12 - 2.2)%	<0.001
**2019****	462,866	2.78 (2.73 - 2.82)%	546,940	2.03 (1.99 - 2.06)%	<0.001
**HFrEF**					
**Overall**	851,481	1.67 (1.65 - 1.7)%	1,013,580	0.66 (0.64 - 0.67)%	<0.001
**2013**	473,540	1.49 (1.46 - 1.53)%	521,841	0.65 (0.63 - 0.68)%	<0.001
**2014**	401,631	1.57 (1.53 - 1.61)%	500,672	0.75 (0.73 - 0.78)%	<0.001
**2015**	402,861	1.62 (1.59 - 1.66)%	508,757	0.74 (0.72 - 0.76)%	<0.001
**2016**	471,965	1.45 (1.41 - 1.48)%	517,255	0.69 (0.67 - 0.71)%	<0.001
**2017**	456,937	1.59 (1.55 - 1.62)%	514,746	0.68 (0.65 - 0.7)%	<0.001
**2018**	435,791	1.62 (1.59 - 1.66)%	520,670	0.79 (0.76 - 0.81)%	<0.001
**2019****	462,866	1.68 (1.64 - 1.71)%	546,940	0.74 (0.72 - 0.77)%	<0.001
**HFpEF**					
**Overall**	851,481	0.64 (0.62 – 0.66)%	1,013,580	1.14 (1.12 - 1.16)%	<0.001
**2013**	473,540	0.63 (0.61 - 0.65)%	521,841	0.90 (0.87 - 0.92)%	<0.001
**2014**	401,631	0.73 (0.71 - 0.76)%	500,672	1.00 (0.97 - 1.03)%	<0.001
**2015**	402,861	0.74 (0.71 - 0.77)%	508,757	1.02 (1.00 - 1.05)%	<0.001
**2016**	471,965	0.65 (0.63 - 0.68)%	517,255	1.00 (0.97 - 1.03)%	<0.001
**2017**	456,937	0.68 (0.66 - 0.71)%	514,746	1.04 (1.01 - 1.06)%	<0.001
**2018**	435,791	0.78 (0.75 - 0.81)%	520,670	1.08 (1.05 - 1.11)%	<0.001
**2019****	462,866	0.76 (0.74 - 0.79)%	546,940	1.01 (0.99 - 1.04)%	<0.001
**HFmrEF**					
**Overall**	851,481	0.10 (0.1 - 0.11)%	1,013,580	0.09 (0.09 - 0.1)%	0.030
**2013**	473,540	0.09 (0.08 - 0.1)%	521,841	0.08 (0.08 - 0.09)%	0.414
**2014**	401,631	0.11 (0.1 - 0.12)%	500,672	0.09 (0.08 - 0.1)%	<0.001
**2015**	402,861	0.10 (0.1 - 0.12)%	508,757	0.09 (0.08 - 0.1)%	0.013
**2016**	471,965	0.09 (0.08 - 0.1)%	517,255	0.09 (0.08 - 0.1)%	0.541
**2017**	456,937	0.11 (0.1 - 0.11)%	514,746	0.10 (0.09 - 0.11)%	0.244
**2018**	435,791	0.12 (0.11 - 0.13)%	520,670	0.11 (0.1 - 0.12)%	0.196
**2019****	462,866	0.11 (0.1 - 0.12)%	546,940	0.09 (0.09 - 0.1)%	0.001
**HFuEF**					
**Overall**	851,481	0.25 (0.24 - 0.26)%	1,013,580	0.17 (0.16 - 0.18)%	<0.001
**2013**	473,540	0.19 (0.18 - 0.2)%	521,841	0.14 (0.13 - 0.15)%	<0.001
**2014**	401,631	0.24 (0.22 - 0.25)%	500,672	0.15 (0.14 - 0.16)%	<0.001
**2015**	402,861	0.24 (0.23 - 0.26)%	508,757	0.17 (0.16 - 0.18)%	<0.001
**2016**	471,965	0.21 (0.2 - 0.22)%	517,255	0.17 (0.16 - 0.18)%	<0.001
**2017**	456,937	0.23 (0.22 - 0.24)%	514,746	0.17 (0.15 - 0.18)%	<0.001
**2018**	435,791	0.26 (0.24 - 0.27)%	520,670	0.19 (0.18 - 0.20)%	<0.001
**2019****	462,866	0.22 (0.21 - 0.23)%	546,940	0.17 (0.16 - 0.19)%	<0.001

Abbreviations: CI = Confidence interval, HF = Heart failure, HFmrEF = Heart failure with mildly reduced ejection fraction, HFpEF = Heart Failure with preserved ejection fraction, HFrEF = Heart failure with reduced ejection fraction, HFuEF = Heart Failure with unknown ejection fraction. * All adult patients alive and enrolled as of the beginning of the calendar year and who have been continuously enrolled during the entire year prior. ** Year 2019 includes until September.

Supplementary Table S3. Baseline characteristics of patients with incident heart failure and mildly reduced left ventricular ejection fraction, stratified by sex (2013–2019).

	HFmrEF Cohort (N =1022)	HFmrEF Men (N =433)	HFmrEF Women (N =589)	P value
**Age at index date (years)**	72.3 ± 18.8	73.6 ± 18.9	71.4 ± 18.6	0.064
**Age groups**				0.607
<45	11.1%	10.4%	11.5%	
45-64	25.6%	23.8%	27.0%	
65-74	14.5%	14.6%	14.4%	
75-84	14.2%	14.1%	14.3%	
≥ 85	34.6%	37.2%	32.8%	
**NYHA class at index date**				0.747
I	12.6%	13.9%	11.7%	
II	44.6%	42.5%	46.2%	
III	37.6%	38.1%	37.2%	
IV	3.5%	3.7%	3.4%	
Unknown	1.7%	1.9%	1.5%	
**Charlson Comorbidity Index**	2.7 ± 1.5	2.8 ± 1.8	2.6 ± 1.5	0.060
**Cardiovascular comorbidities**				
**Hypertension**	61.3%	64.2%	59.1%	0.097
**Dyslipidaemia**	44.9%	43.0%	46.4%	0.281
**Diabetes type 1**	4.4%	4.4%	4.4%	0.984
**Diabetes type 2**	27.1%	26.6%	27.5%	0.737
**Atrial fibrillation**	29.8%	30.3%	29.4%	0.760
**Coronary artery disease**	31.1%	31.2%	31.1%	0.971
**Peripheral arterial disease**	3.0%	2.5%	3.4%	0.431
**Other comorbidities**				
**Stroke**	9.3%	10.2%	8.7%	0.414
**Chronic kidney disease**	30.3%	30.3%	30.4%	0.479
**Stage unknown**	12.5%	13.6%	11.7%	
**Stage I**	0.5%	0.5%	0.5%	
**Stage II**	2.9%	3.2%	2.7%	
**Stage III**	11.4%	11.3%	11.4%	
**Stage IV**	1.8%	1.2%	2.2%	
**End stage**	1.1%	0.7%	1.7%	
**COPD**	9.5%	10.9%	8.5%	0.202
**Asthma**	10.6%	11.1%	10.2%	0.644
**Anemia**	27.3%	28.9%	26.2%	0.334
**Hepatic disease**	3.6%	4.4%	3.1%	0.260
**Malignant neoplasm**	11.5%	11.1%	11.7%	0.755

Abbreviations: COPD = Chronic obstructive pulmonary disease, NYHA = New York Heart Association, LVEF = Left ventricular ejection fraction, HF = Heart failure, HFmrEF = Heart failure with mildly reduced ejection fraction, HFpEF = Heart failure with preserved ejection fraction, HFrEF = Heart failure with reduced ejection fraction, HFuEF = Heart Failure with unknown ejection fraction.

Supplementary Table S4. Baseline characteristics of patients with incident heart failure and unknown left ventricular ejection fraction, stratified by sex (2013–2019).

	HFuEF Cohort (N = 5017)	HFuEF Men (N = 2807)	HFuEF Women (N = 2210)	P value
**Age at index date (years)**	72.3 ± 18.9	72.5 ± 18.8	72.2 ± 18.9	0.576
**Age groups**				0.272
<45	10.1%	9.8%	10.4%	
45-64	27.6%	27.5%	27.7%	
65-74	13.5%	13.3%	13.8%	
75-84	14.1%	15.0%	12.9%	
≥ 85	34.8%	34.5%	35.3%	
**NYHA class at index date**				0.931
I	14.2%	14.3%	14.0%	
II	39.5%	39.1%	40.1%	
III	42.0%	42.3%	41.7%	
IV	2.4%	2.3%	2.4%	
Unknown	1.9%	2.0%	1.8%	
**Charlson Comorbidity Index**	2.7 ± 1.6	2.7 ± 1.8	2.6 ± 1.6	0.038
**Cardiovascular comorbidities**				
**Hypertension**	58.3%	57.9%	59.0%	0.431
**Dyslipidaemia**	47.4%	47.9%	46.8%	0.427
**Diabetes type 1**	3.4%	3.3%	3.5%	0.675
**Diabetes type 2**	28.3%	28.8%	27.8%	0.451
**Atrial fibrillation**	28.5%	28.0%	29.1%	0.394
**Coronary artery disease**	30.8%	31.5%	30.1%	0.282
**Peripheral arterial disease**	6.0%	6.1%	6.0%	0.860
**Other comorbidities**				
**Stroke**	10.7%	11.5%	9.6%	0.023
**Chronic kidney disease**	23.4%	24.2%	22.3%	0.810
**Stage unknown**	9.6%	10.2%	8.9%	
**Stage I**	0.5%	0.5%	0.6%	
**Stage II**	2.3%	2.5%	2.0%	
**Stage III**	8.0%	8.2%	7.8%	
**Stage IV**	2.3%	2.2%	2.4%	
**End stage**	0.6%	0.6%	0.5%	
**COPD**	12.6%	13.0%	12.1%	0.353
**Asthma**	8.7%	8.0%	9.5%	0.063
**Anemia**	24.3%	24.4%	24.1%	0.786
**Hepatic disease**	3.8%	4.2%	3.3%	0.098
**Malignant neoplasm**	12.3%	11.9%	12.8%	0.356

Abbreviations: COPD = Chronic obstructive pulmonary disease, NYHA = New York Heart Association, LVEF = Left ventricular ejection fraction, HF = Heart failure, HFmrEF = Heart failure with mildly reduced ejection fraction, HFpEF = Heart failure with preserved ejection fraction, HFrEF = Heart failure with reduced ejection fraction, HFuEF = Heart Failure with unknown ejection fraction.
